# Orthogonal Design Optimisation of the Sintering Process for MnZn Ferrites with Step-Sintering Verification

**DOI:** 10.3390/ma19040779

**Published:** 2026-02-16

**Authors:** Mengrui Li, Shuyu Sun, Boon Xian Chai, Yuqi Wang, M. Akbar Rhamdhani, Shanqing Xu, Li Wang

**Affiliations:** 1 School of Mechanical, Electrical and Information Engineering, Shandong University, Weihai 264209, China; maureenli@swin.edu.au (M.L.); 202337667@mail.sdu.edu.cn (S.S.); 2 School of Engineering, Swinburne University of Technology, Hawthorn, VIC 3122, Australia; 3 School of Electromechanical and Automotive Engineering, Yantai University, Yantai 264005, China

**Keywords:** MnZn ferrites, sintering process optimisation, orthogonal design, initial permeability, power loss

## Abstract

MnZn ferrites for power electronics require a well-controlled sintering window to balance high initial permeability (*µ_i_*) with low power loss (*P_cv_*). Here, an L9 (3^3^) orthogonal design was employed to quantify the main effects of sintering temperature, holding time, and oxygen partial pressure on *µ_i_* and *P_cv_* within the investigated processing window, enabling rapid mapping of feasible sintering windows. The orthogonal analysis identifies the relative significance of each factor and reveals a clear performance trade-off between *µ_i_* and *P_cv_*. For maximising *µ_i_*, the optimal sintering condition was 1250 °C, 4 h holding time, and 3.5% oxygen partial pressure, yielding a *µ_i_* of 3453 and a *P_cv_* of 466 mW/cm^3^ at 100 kHz/200 mT. For minimising *P_cv_*, the optimal condition was 1250 °C, 3.5 h holding time, and 5% oxygen partial pressure, resulting in a *µ_i_* of 2678, with *P_cv_* of 400 mW/cm^3^ at 100 kHz/200 mT and 182 mW/cm^3^ at 500 kHz/50 mT. Targeted verification together with XRD, SEM grain-size statistics, and magnetic-loss separation were used to strengthen the process-structure-property interpretation. Overall, the orthogonal-screening-plus-verification strategy provides a practical framework for predicting application-relevant performance trends of MnZn ferrites within a defined processing window.

## 1. Introduction

The transition to renewable and greener energy sources, along with the growing adoption of applications, such as electric vehicles, has increased the demand for energy storage systems and associated devices, including batteries and chargers, that offer greater miniaturisation and higher efficiency [1,2,3]. Battery chargers contain a variety of electrical components, such as inductors, transformers, and sensors, many of which employ MnZn ferrites as the magnetic core material. The properties of MnZn ferrites directly influence the performance of these electrical components and, in turn, affect the overall efficiency and reliability of battery chargers. Consequently, enhancing the performance of MnZn ferrites, particularly achieving high initial permeability (*µ_i_*), low power loss (*P_cv_*), and high saturation magnetic flux density (*B_s_*), has become a key priority to meet the requirements of emerging technologies and new application fields [4,5]. Among the various fabrication techniques, the oxide ceramic method is one of the most widely adopted production processes in industry, preferred over alternative approaches, such as sol-gel and coprecipitation methods, due to its scalability and cost-effectiveness. Within this process, sintering is a critical stage, as it plays a decisive role in determining the comprehensive performance of MnZn ferrites through phase transformations and grain growth that occur during this step [6,7].

To date, many studies have been conducted to optimise the sintering process parameters to produce MnZn ferrites with improved magnetic properties for various working conditions. In 2012, Reddy et al. [8] investigated the effect of sintering temperature on MnZn ferrites’ properties and found that increasing the sintering temperature reduced interatomic distances, resulting in higher material density. Denser MnZn ferrites contain fewer pores and defects, which enhances initial permeability and suppresses hysteresis loss (*P_h_*) [9]. Liu et al. [10] reported on the influence of oxygen partial pressure ($P_{O_{2}}$) during sintering on the core loss (*P_cv_*) of MnZn ferrites. Samples sintered at $P_{O_{2}}$ values of 0.75% and 1.90% exhibited low *P_cv_* values of 275 mW/cm^3^ at 100 kHz and 200 mT. At low frequencies, *P_cv_* can be separated into hysteresis loss (*P_h_*) and eddy current loss (*P_e_*). Loss separation analysis showed that both *P_h_* and *P_e_* decreased with increasing $P_{O_{2}}$. This was attributed to the creation of more cation vacancies at higher $P_{O_{2}}$, which promoted the migration of dopants, such as Ca and Si, to grain boundaries. This increased grain boundary resistivity, reducing *P_e_*, while also lowering defect levels within grains, which in turn reduced *P_h_*. In 2022, Priya et al. [11] examined the effect of heat treatment on the structural, morphological, dielectric, and magnetic properties of Mg-Zn ferrite nanoparticles. M-H loop analysis indicated that saturation magnetization (*M_s_*) increased with sintering temperature. In spinel ferrites, magnetization arises from the difference between the magnetizations (*M_B_* and *M_A_*) of the octahedral and tetrahedral sites. Higher sintering temperatures caused Zn^2+^ (0 µ_B_) ions to migrate from B-sites to A-sites, forcing Fe^3+^ (5 µ_B_) ions into B-sites, thereby increasing B-site magnetization and raising *M_s_*. Tsakaloudi et al. [12] developed a fast sintering process for MnZn ferrites (*µ_i_* > 5000) by optimizing raw material morphology, In_2_O_3_ doping, sintering atmosphere, and cooling rate. This approach shortened the sintering cycle by 34%, increased initial permeability by 35%, and reduced magnetic losses by 14–34% across a range of frequencies. Collectively, these studies confirm that the sintering process plays a critical role in determining the performance of soft magnetic ferrites.

It is widely recognised that the sintering process comprises three stages: the heating period, the sintering period, and the cooling period, each governed by specific parameters. In the sintering period in particular, temperature, duration, and atmosphere are the primary factors, and together they directly influence the microstructure and, consequently, the overall properties of MnZn ferrites. However, there has been limited research assessing which of these parameters plays the most critical role in determining the comprehensive performance of MnZn ferrites. In particular, many optimisation studies focus on single-factor tuning or a single performance metric, and the resulting trends are often difficult to translate into an actionable processing window under application-relevant excitation conditions. As a result, a practical process-property map that explicitly balances permeability and power loss within a defined parameter space is still lacking. Furthermore, most studies have not adequately addressed the connection between MnZn ferrites and their application in electrical components. In some cases, investigations have reported only the final performance of MnZn ferrites without analysing the reactions and transformations that occur during sintering. Given the increasingly stringent requirements of emerging technologies and applications, the development of suitable and effective experimental designs to optimise the sintering process is both urgent and meaningful for producing MnZn ferrites with superior performance. In addition, carefully planned qualitative experiments are essential to fully elucidate the mechanisms of the sintering process.

The key contribution of this study is to convert orthogonal screening into a practically usable, application-oriented sintering-window map, supported by verification and mechanistic process-path evidence. In this work, an L9 orthogonal design was employed to evaluate the main effects of sintering temperature, holding time and oxygen partial pressure on the initial permeability (*µ_i_*) and power loss (*P_cv_*) of MnZn ferrites under the targeted operating conditions. Based on the main-effects ranking, a data-driven process-window map was established to guide feasible routes for either *µ_i_*-maximised or loss-minimised performance, thereby making the *µ_i_*-*P_cv_* trade-off explicit within the investigated range. To ensure reliable interpretation beyond parameter screening, the proposed windows were further validated and mechanistically rationalised through systematic structure-property characterisation, linking atmosphere/temperature/time to phase constitution, densification, and microstructure, and ultimately to magnetic-loss behaviour.

## 2. Methodology

### 2.1. Materials and Equipment

**Figure 1:**
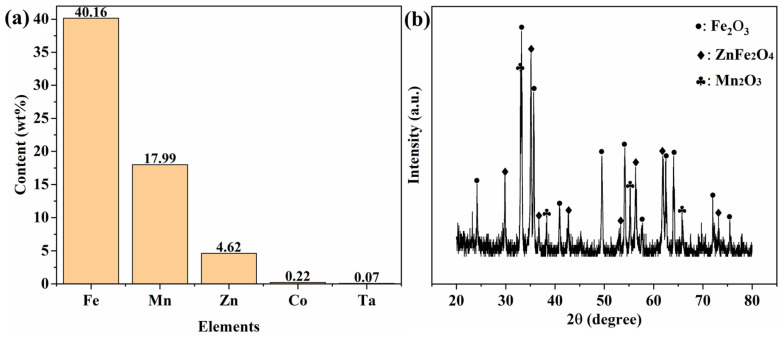
(**a**) ICP-measured elemental contents (wt%) of the as-received GP95 powders and (**b**) XRD pattern of the as-received GP95 powders.

Commercial pre-fired MnZn ferrite powders (product model GP95) used in this study were sourced from Nantong Guanyouda Magnet Co., Ltd. (GYD) (Jiangsu, China), a manufacturer specialising in MnZn ferrite powders and magnetic cores. Their elemental and phase compositions are shown in Figure 1. ICP analysis (Figure 1a) gives Fe = 40.16 wt%, Mn = 17.99 wt%, Zn = 4.62 wt%, with Co and Ta present as dopants at 0.22 wt% and 0.07 wt%, respectively (as-received powders). Figure 1b shows diffraction peaks from Fe_2_O_3_ and Mn_2_O_3_, indicating that the as-received GP95 is a partially reacted, pre-fired powder rather than a fully reacted single-phase ferrite ceramic. This is consistent with supplier-reported pre-sintering at 800–1050 °C. These temperatures are typically sufficient to form some ZnFe_2_O_4_, whose formation begins about 700 °C, but may be insufficient to complete conversion to the MnZn ferrite spinel. The dopants, likely present as Co_2_O_3_ and Ta_2_O_5_, were not detected by X-ray diffraction because their concentrations are extremely low and may be below the detection limit of laboratory XRD. For reproducibility, the above composition and key supplier specifications are also summarised in the Supplementary Information (Table S1).

**Figure 2:**
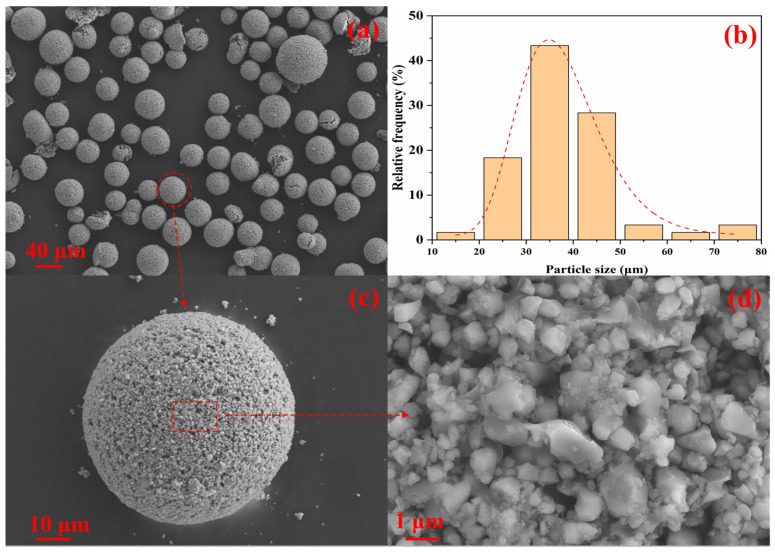
SEM images of (**a**) GYD magnetic particles morphology, (**b**) particle size distribution, (**c**) single particle morphology, and (**d**) magnetic aggregation in a particle.

The powder morphology is shown in Figure 2a. The particles are predominantly spherical, with a size distribution approximately from 38 to 75 µm as quantified in Figure 2b,c. At higher magnification, as shown in Figure 2d, numerous irregular fragments are observed to adhere to or be embedded within the spheres. Such granulated, near-spherical morphology is consistent with the conventional oxide ceramic process used for commercial soft ferrite powder. Weighed oxides (typically Fe_2_O_3_, Mn_3_O_4_, and ZnO) are milled to homogenise composition, pre-fired in a furnace at 800–1050 °C, then re-milled with specific additives. The slurry is subsequently spray-granulated, during which a polyvinyl alcohol (PVA) binder is added so that agglomerates consolidate into spherical particles.

Two main types of equipment were used in this work. Green bodies were formed using a hydraulic press, which is commonly employed for powder compaction and shaping. Sintering was carried out in a bell-type vacuum/controlled-atmosphere furnace equipped with MoSi_2_ heating elements (maximum 1500 °C), enabling O_2_/N_2_ gas mixtures or inert atmosphere to be introduced, as well as vacuum operation. In this study, the GP95 powders were pressed into toroidal specimens of defined dimensions under a pressure of 400 MPa using the hydraulic press and subsequently sintered under different sintering conditions in the bell-type vacuum atmosphere sintering furnace, following the orthogonal experimental design.

### 2.2. Characterisation

Phase composition of the sintered samples was analysed by X-ray diffraction (XRD, Rigaku Ultima IV, Japan) using Cu *K_α_* radiation (*λ* = 1.5406 Å) in the 2θ range from 10° to 80° at room temperature, and Rietveld refinement was performed using TOPAS. For microstructural observation, toroidal specimens were fractured mechanically, and the fracture and surface morphologies were examined by scanning electron microscopy (SEM, ZEISS Gemini SEM 300, Jena, Germany). Grain sizes were measured from SEM micrographs using the Nano-Measurer 1.2 software, and the mean grain size was calculated accordingly. To improve statistical rigour, grain size was determined from at least three randomly selected fields of view, with a minimum of 100 grains per sample. Crystal orientation and grain-boundary character were assessed by electron backscatter diffraction (EBSD, EDAX-TSL, Mahwah, NJ, USA) after standard mechanical polishing (including a final fine polishing step). Thermogravimetric analysis (TGA) was performed on a NETZSCH STA 449 F3 Jupiter (NETZSCH, Selb, Germany) under flowing N_2_ atmosphere from room temperature to 1200 °C at a heating rate of 5 °C/min using a sample mass of ~10 mg. Bulk density was measured by the Archimedes method using a density tester (LSD-300, Xiamen, Fujian, China). Electrical resistivity was determined using a four-probe resistivity tester (ST2722-SD, Suzhou, Jiangsu, China). The initial permeability was calculated from the inductance measured with an LCR impedance analyser (LCR-8210, Suzhou, Jiangsu, China). Power loss was measured using a B-H analyser (MATS-3000SA/1M, Loudi, Hunan, China).

### 2.3. Orthogonal Experiment Design

**Figure 3:**
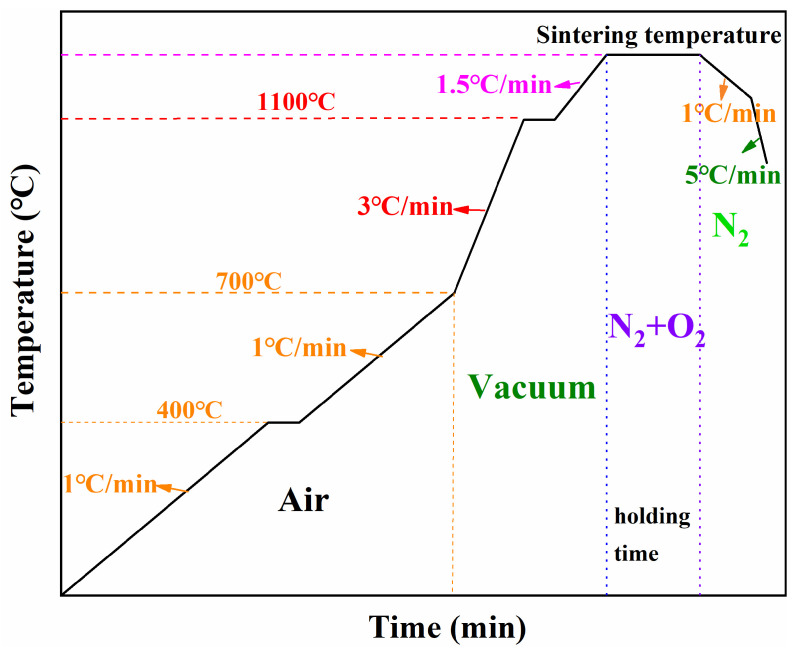
Temperature and atmosphere history for the sintering process. Sintering temperature includes 1250 °C, 1280 °C, and 1310 °C, and holding time includes 3 h, 3.5 h, and 4 h. The oxygen partial pressure includes 2%, 5%, and 3.5%.

This study focused on the isothermal holding stage of the sintering process. To aid interpretation, the temperature diagram of the full sintering process is provided in Figure 3, showing three primary stages: heating, holding, and cooling, with the associated parameters annotated. The holding stage was the principal subject of investigation because it encompasses multiple controllable variables, including sintering temperature, holding time, and oxygen partial pressure. These process variables strongly influence the microstructure and, consequently, the overall performance of MnZn ferrites.

The sintering process for MnZn ferrites comprises three main stages and multiple interacting parameters, which complicates experimental design and increases workload. Therefore, an L9 orthogonal design was adopted as a screening strategy to evaluate the main effects of key holding-stage parameters with a limited number of experiments. This approach enables rapid identification of dominant factors and feasible process windows from a reduced experimental matrix, thereby improving the efficiency of process optimisation [13,14,15]. The L9 orthogonal array primarily captures main effects, and potential interaction effects are not exhaustively resolved and are therefore treated as a secondary consideration within the scope of this screening design. However, temperature and oxygen partial pressure may still interact through coupled defect equilibration and diffusion/volatilisation kinetics during sintering, potentially shifting the oxygen level that yields optimal microstructure and magnetic performance at different temperatures. A rigorous quantification of such interactions would require an expanded design, which is beyond the scope of the present screening study.

Sintering temperature, holding time, and oxygen partial pressure are critical factors that strongly influence the performance of MnZn ferrites. Guided by the literature [16,17,18,19,20] and preliminary temperature screening to define the feasible sintering temperature window (summarised in Table S2), three levels were selected for each factor: temperature at 1250 °C, 1280 °C, and 1310 °C; oxygen partial pressure at 2.0%, 3.5%, and 5.0%, and holding time at 3.0 h, 3.5 h, and 4.0 h. The oxygen partial pressure levels (2.0–5.0% O_2_ in N_2_) were chosen to bracket a practical and controlled processing window for MnZn ferrite sintering, ensuring stable densification while avoiding excessively reducing or overly oxidising conditions. The lower (2.0%) and upper (5.0%) bounds represent the feasible limits in this work, and 3.5% was included as an intermediate level to capture potential non-monotonic responses of magnetic properties to the atmosphere. An L9 (3^3^) orthogonal array (three factors at three levels, nine experimental runs) was generated using Orthogonal Design Assistant 3.1 software, as listed in Table 1. The response variables were the initial permeability (*µ_i_*) and the power loss (*P_cv_*) measured under the targeted operating conditions, and the factor significance was evaluated and discussed in Section 3.2 and Section 3.3. During sintering, the desired oxygen partial pressure levels were implemented by supplying premixed O_2_/N_2_ gases.

## 3. Results and Discussion

### 3.1. Phase Constitution and Lattice Parameter

**Figure 4:**
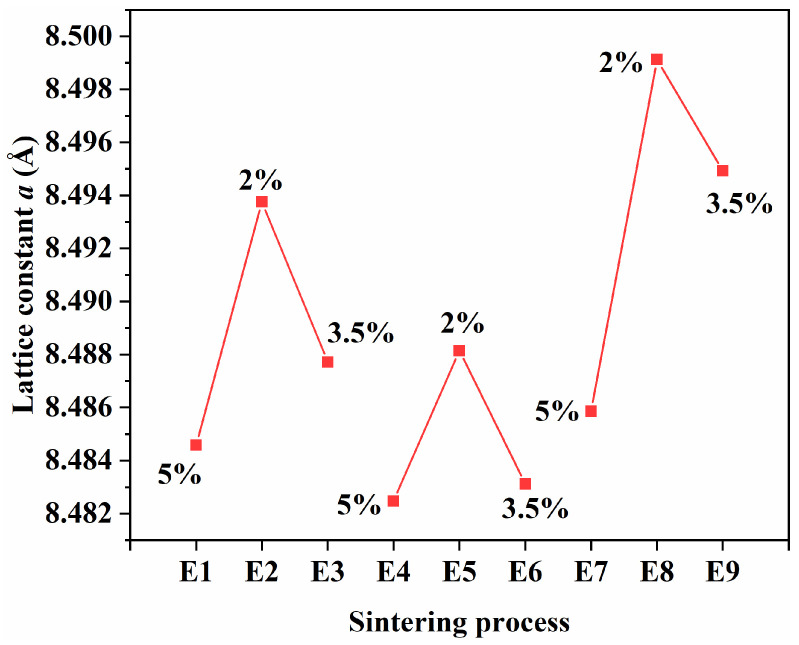
Estimated lattice constant *a* of MnZn ferrite (E1–E9) under different oxygen partial pressures during sintering (XRD patterns are provided in Figure S1).

Figure 4 illustrates the variation in the estimated lattice constant *a* under different sintering atmospheres. It is evident that the lattice parameter increases as the oxygen partial pressure decreases from 5% to 2%. For example, samples sintered at 2% O_2_ (E2 and E8) show the highest lattice constants of 8.495 Å and 8.499 Å, respectively, which are slightly higher than those of samples sintered at 5% O_2_ (~8.485 Å). The lattice parameters for the orthogonal set (E1–E9) were derived from XRD peak positions of the spinel phase and are used here to compare relative trends. The corresponding XRD patterns are provided in the Supplementary Information (Figure S1). This trend may be associated with atmosphere-dependent oxygen non-stoichiometry and defect chemistry in MnZn spinel ferrite. Under a lower $P_{O_{2}}$, changes in defect concentration (e.g., oxygen vacancies) and redox equilibrium may lead to a slight lattice expansion, whereas higher $P_{O_{2}}$ tends to suppress such effects and can result in a marginal lattice contraction [21,22]. Because the valence states (e.g., Fe^2+^/Fe^3+^) were not quantitatively determined for all E1–E9 samples, the above explanation is provided as a plausible interpretation rather than a definitive mechanism. Minor variations among the three orthogonal groups may also arise from temperature differences, holding time, or Zn volatilisation during sintering. Overall, the XRD results and lattice-parameter trends indicate that oxygen partial pressure plays an important role in influencing the crystal structure of MnZn ferrites within the investigated processing window.

### 3.2. Initial Permeability

Initial permeability *µ_i_* is a key performance indicator for MnZn ferrites, reflecting the material’s ability to respond to an applied magnetic field. To identify processing routes that enhance *µ_i_*, an orthogonal analysis was performed on samples produced under an L9 design as a main-effects screening strategy. The results are summarised in Table 2. For each factor, *K* denotes the mean *µ_i_* at a given level (*K*_1_, *K*_2_, *K*_3_ for temperature levels), and *R* is the range for that factor, defined as the maximum minus the minimum level average (*R* = max *K_i_* − min *K_i_*). The *R* value, therefore, indicates the influence of a factor on *µ_i_*, whereas a larger *K* at a specific level identifies the preferred level for that factor within the investigated parameter window. In Table 2, the relation R(B) > R(A) > R(C) indicates that factor B, namely the oxygen partial pressure, exerts the greatest impact on the initial permeability *µ_i_*. This ranking is further supported by a main-effects ANOVA of the L9 dataset (Supplementary Information, Table S3), which confirms that oxygen partial pressure contributes most strongly to *µ_i_*, followed by sintering temperature, while holding time has the smallest contribution within the investigated window. Meanwhile, the *K* value reaches its maximum when factor A is at level 1, factor B is at level 3, and factor C is at level 3. Accordingly, it can be inferred that MnZn ferrites are more likely to achieve a higher *µ_i_* under the A1B3C3 condition, corresponding to a sintering temperature of 1250 °C, an oxygen partial pressure of 3.5%, and a holding time of 4 h. This inference is consistent with the experimental result for sample E3, which exhibited a remarkably high initial permeability of 3453 compared with other samples in the orthogonal experiment, supporting the validity of the orthogonal screening. The L9 design primarily captures main effects, while a more rigorous quantification of interaction and statistical uncertainty would require additional runs and repeats. Such temperature–oxygen interactions may arise because temperature accelerates defect equilibration and diffusion kinetics, thereby strengthening or weakening the influence of oxygen partial pressure on oxygen non-stoichiometry, grain-boundary resistivity, and ultimately permeability and power loss.

In summary, the relative importance of the three factors affecting *µ_i_* can be ranked as: oxygen partial pressure > sintering temperature > holding time. Consistent with this ranking, *µ_i_* reached a maximum at an intermediate oxygen partial pressure (3.5% $P_{O_{2}}$) compared with 2% or 5% $P_{O_{2}}$. In MnZn spinel ferrites, the sintering atmosphere may modify the defect chemistry and redox equilibrium, which can affect magnetic softness through changes in magnetocrystalline anisotropy, magnetostriction and internal stress. However, because *M_s_*, *K_1_*, and the Fe^2+^/Fe^3+^ ratio were not quantitatively determined for all E1–E9 samples, the atmosphere-related interactions in this work are qualitative, and we therefore focus on experimentally observable structure–property correlations.

**Figure 5:**
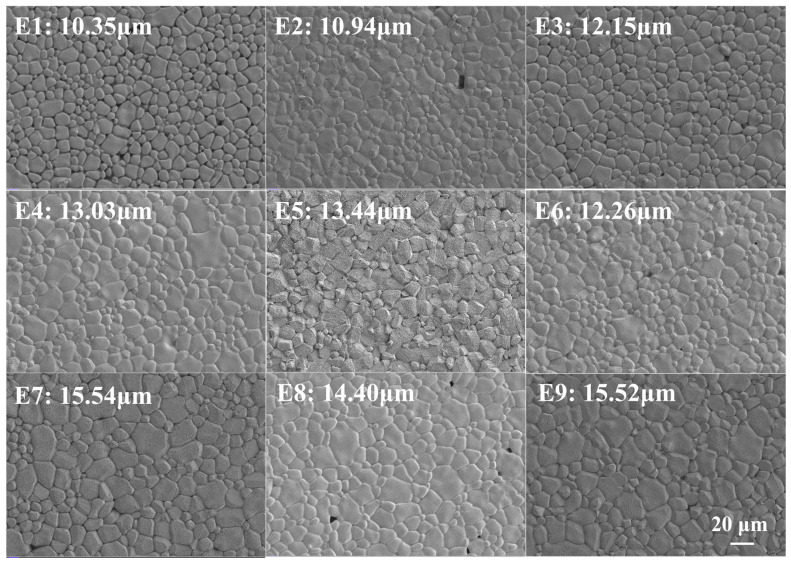
SEM micrographs with average grain sizes (same scale bar: 20 µm) of MnZn ferrites (E1–E9) prepared under the L9 orthogonal sintering design.

**Figure 6:**
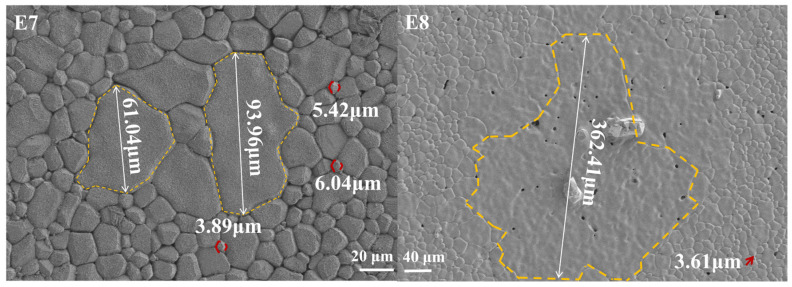
SEM micrographs showing abnormal grain growth in MnZn ferrites (E7 and E8) sintered at 1310 °C (the yellow lines cirlcle the abnormal grains).

Except for oxygen partial pressure, the sintering temperature also plays an essential role in determining the initial permeability (*µ_i_*) of MnZn ferrites. Within the investigated L9 parameter window, the *µ_i_* of samples sintered at 1250 °C tended to be higher than that of samples sintered at 1280 °C and 1310 °C. This trend can be attributed to the fact that the sintering temperature not only provides the energy required for solid-phase reactions but also influences the microstructure of MnZn ferrites, including grain morphology, grain size, and density. As shown in Figure 5, the samples sintered at 1250 °C exhibited a more uniform microstructure with clearer grain boundaries compared with those sintered at 1280 °C and 1310 °C, which is favourable for domain-wall motion and can reduce pinning sites [23]. Since the initial permeability is the combined contribution of domain wall movement and spin rotation, expressed as $\mu_{i}=\mu_{irot}+\mu_{imov}$, such uniformity and reduced defects are conducive to enhancing *µ_i_*. However, as the sintering temperature further increased, grain coarsening occurred, leading to the formation of abnormal grains (Figure 6) and a broader grain size distribution. This microstructural heterogeneity is expected to increase pinning and internal stress, consistent with reduced magnetic softness (e.g., higher coercivity) and therefore lower *µ_i_* [24]. Therefore, an appropriately lower sintering temperature (e.g., 1250 °C) is beneficial for achieving higher *µ_i_* in the present study.

### 3.3. Power Loss

To minimise the power loss (*P_cv_*) under the targeted operating conditions, an orthogonal analysis was carried out using the measured *P_cv_* values at 100 kHz/200 mT (Table 3) and 500 kHz/50 mT (Table S4) for the L9 sample set. The analysis procedure follows that used for *µ_i_*, where *K* denotes the mean response at each factor level, and *R* represents the range (*R =* max *K_i_* − min *K_i_*). A larger *R* value indicates that the corresponding factor exerts a stronger influence on *P_cv_* within the investigated parameter window. The results showed that the oxygen partial pressure was the most significant factor affecting *P_cv_* during sintering, followed by the sintering temperature, while the holding time had the least effect. This ranking is further supported by a main-effects ANOVA of the L9 dataset at 100 kHz/200 mT (Supplementary Information, Table S5), which confirms that oxygen partial pressure and temperature contribute more strongly to *P_cv_* than holding time within the investigated window. This ranking differs from that for *µ_i_*, reflecting the different sensitivities of permeability and loss to processing variables and the trade-off between performance metrics.

For *P_cv_*, the optimum level corresponds to the minimum *K* value since the experimental objective was to minimise power loss. This criterion was also applicable to the *P_cv_* data at 500 kHz/50 mT. Importantly, the minimum-loss sample depends on the excitation condition. The lowest *P_cv_* at 100 kHz/200 mT was obtained for E4 (1280 °C, 5% $P_{O_{2}}$, and 3.5 h), whereas at 500 kHz/50 mT the minimum *P_cv_* was achieved by E1 (1250 °C, 5% $P_{O_{2}}$, and 3 h) (Table S4). This observation highlights that the optimal sintering condition should be selected based on the targeted operating points and the required balance between *µ_i_* and *P_cv_*. Nevertheless, the orthogonal analysis consistently indicates that a higher oxygen partial pressure (5% $P_{O_{2}}$) is favourable for reducing *P_cv_* within the investigated window.

**Figure 7:**
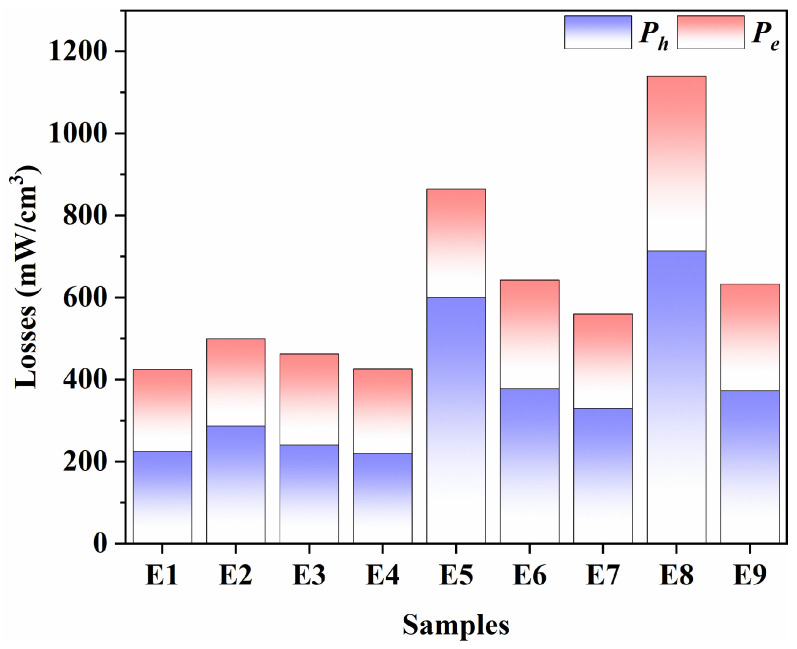
Loss separation of MnZn ferrite samples (E1–E9) measured at 100 kHz/200 mT, showing the decomposition of total power loss *P_cv_* into hysteresis loss *P_h_* and eddy-current loss *P_e_*.

At the operating condition of this work (100 kHz/200 mT), loss separation was further performed for all samples (E1–E9) to clarify the origin of the variations in the total power loss *P_cv_*. As shown in Figure 7, *P_cv_* is decomposed into the *P_h_* and *P_e_* under the same measurement conditions. A key mechanistic outcome is that, at 100 kHz/200 mT, the differences in *P_cv_* across the L9 set are predominantly *P_h_*-driven. Specifically, E4 exhibited the minimum *P_cv_* mainly because *P_h_* is markedly reduced, whereas *P_e_* changes only modestly. This *P_h_*-dominant behaviour is consistent with the microstructural features associated with magnetic softness, where reduced domain-wall pinning (e.g., fewer pores or defects), together with a more uniform grain-size distribution and lower porosity, suppresses irreversible magnetisation dissipation. Overall, the loss-separation provides direct, mechanism-level evidence that the minimum-loss case (E4) is governed primarily by *P_h_* reduction.

Consistent with the factor ranking, increasing the oxygen partial pressure to 5% provides a robust route to reduce *P_cv_* in the present processing window. In practice, the sintering atmosphere can influence defect chemistry and oxidation equilibria and thereby affect microstructural development and magnetic softness. These defect-chemistry-related interpretations are qualitative in the present work, as oxygen non-stoichiometry and Fe^2+^/Fe^3+^ equilibrium were not directly quantified for the full L9 sample set. The present loss-separation result further shows that, at 100 kHz/200 mT, the reduction in *P_cv_* is mainly driven by a decrease in *P_h_*, while *P_e_* plays a secondary role in differentiating the total loss among E1–E9. In addition, *P_e_* generally correlates with electrical resistivity and characteristic eddy-current path length, which depend on densification and grain size. However, in the current dataset, *P_e_* is not the dominant contribution at 100 kHz/200 mT.

(1) $$P_{cv}=P_{h}+P_{e}+P_{r}=K_{h}B_{m}^{3}f+K_{e}B_{m}^{2}f^{2}D^{2}/\rho +P_{r}$$

where *K_h_* and *K_e_* are loss-related constants, *f* is the excitation frequency, *B_m_* is the peak flux density, *D* is the grain size, and *ρ* is the electrical resistivity. At 100 kHz/200 mT, *P_h_* constitutes a major fraction of *P_cv_* for these soft ferrites, consistent with the loss-separation results. Therefore, processing routes that improve magnetic softness (e.g., reduced pinning and improved microstructural uniformity) are expected to effectively suppress *P_h_*.

Another important aspect of the sintering temperature is its impact on *P_e_* through microstructural evolution. As previously discussed, the grain size of MnZn ferrites sintered at 1250 °C was generally smaller than that of samples sintered at higher temperatures, thereby reducing *P_e_* according to Equation (1). However, with increasing sintering temperature, grain growth became more pronounced and abnormal grain growth may occur, which can unfavourably affect both *P_e_* and magnetic softness. In summary, an appropriate combination of oxygen partial pressure and sintering temperature is essential for reducing *P_cv_* under the targeted operating conditions.

Given that holding time shows the smallest *R* value in the orthogonal analysis, its effect on *P_cv_* is secondary within the 3–4 h range investigated here and is therefore not over-interpreted. Additionally, since the orthogonal analysis suggests a preference for a lower temperature level within the investigated window, it raises the question of whether further lowering the sintering temperature would continue to improve performance. To verify the validity of 1250 °C as the optimum sintering temperature, toroidal samples were sintered at 1230 °C, with a holding time of 3.5 h under 5% oxygen partial pressure, and the results are presented in Table S6. It was observed that both *µ_i_* and *P_cv_* deteriorated when the sintering temperature decreased, which may be attributed to the insufficient thermal driving force for adequate microstructural development and magnetic softness. This result supports the selection of 1250 °C as a practical low-loss temperature within the investigated window, particularly when performance at higher frequency (500 kHz/50 mT) is prioritised.

### 3.4. Sintering Optimisation

**Figure 8:**
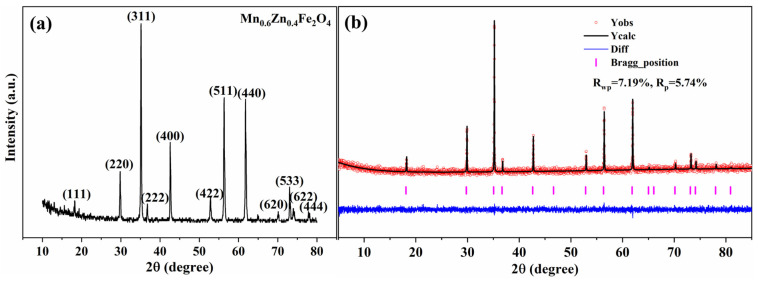
(**a**) XRD pattern, and (**b**) Rietveld refinement of the MnZn ferrite sintered under the optimised scheme (1250 °C, 5% $P_{O_{2}}$, and 3.5 h).

A verification sample was prepared independently from the L9 set using a representative condition from the low-loss window suggested by orthogonal screening (1250 °C, 5% $P_{O_{2}}$, and 3.5 h). This condition retains the preferred high oxygen partial pressure for loss reduction and the low-temperature tendency within the investigated window, while the holding time was set at the middle level, given its minor main effect over 3–4 h. The sample exhibits *µ_i_* = 2678 and low power losses of 400 mW/cm^3^ at 100 kHz/200 mT and 182 mW/cm^3^ at 500 kHz/50 mT, supporting the orthogonal conclusions and confirming that this process window provides a favourable balance between permeability and loss over the targeted frequency range. Duplicate preparations of this optimised low-loss condition show good reproducibility, as summarised in Table S7. The XRD analysis (Figure 8a) shows that all diffraction peaks can be indexed to the cubic spinel Mn_0.6_Zn_0.4_Fe_2_O_4_ phase (PDF#74-2401), and no additional reflections attributable to secondary phases are observed within the detection limit of laboratory XRD. To further strengthen the reliability of the phase and lattice analysis, Rietveld refinement was performed for this representative sample (Figure 8b). The calculated profile matches the experimental pattern well, with *R_wp_* = 7.19% and *R_p_* = 5.74%, a flat difference curve, and correctly located Bragg positions, supporting the formation of a well-crystallised single-phase spinel under the selected sintering scheme. These results provide a solid structural basis for the subsequent discussion of magnetic properties.

**Figure 9:**
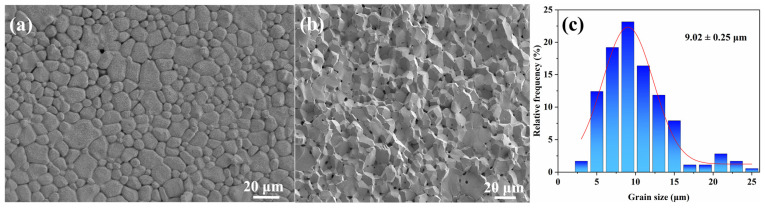
SEM images of the optimised MnZn ferrite (1250 °C, 5% $P_{O_{2}}$, and 3.5 h): (**a**) surface microstructure, (**b**) fracture surface, and (**c**) grain size distribution (mean = 9.02 ± 0.25 µm).

To elucidate the microstructural origin of the low-loss behaviour, the optimised sample was examined by SEM, and the grain size was quantified using Nano-Measurer (Figure 9). The micrographs show a relatively uniform polycrystalline microstructure with clear grain boundaries and no obvious abnormal grain growth. Importantly, the grain-size distribution (Figure 9c) is narrow and shows a single main peak, giving an average grain size of 9.02 ± 0.25 µm, supporting good microstructural homogeneity under the selected sintering condition. In the frequency range investigated here (100–500 kHz), the total power loss is commonly discussed in terms of the hysteresis- and eddy-current-related contributions, and their relative importance depends on both excitation conditions and microstructural and stress-related factors [25,26,27]. At 100 kHz/200 mT, *P_cv_* is primarily associated with a decrease in *P_h_*, whereas the change in *P_e_* is comparatively less pronounced. The improved grain-size uniformity observed in Figure 9 is consistent with reduced domain-wall pinning and reduced hysteresis-related dissipation, as reported for fine and homogeneous microstructure in low-loss MnZn ferrites [25,28,29,30]. In addition, the optimised sample shows a modest increase in electrical resistivity (from 24.692 to 26.647 Ω·m), which is expected to further suppress the eddy-current contribution according to Equation (1). Overall, the SEM observations, together with the resistivity measurement and loss separation, provide an experimentally supported explanation for the reduced *P_cv_* achieved under the optimised sintering scheme [31,32].

**Figure 10:**
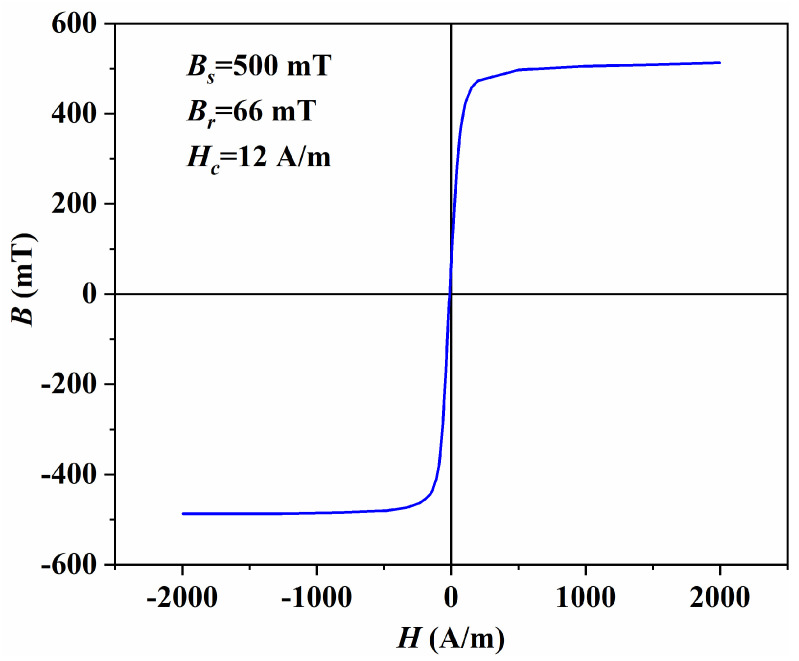
*B*-*H* hysteresis loop of MnZn ferrites sintered under optimised sintering scheme.

Figure 10 shows the *B*-*H* hysteresis loop of the MnZn ferrites prepared under the optimised sintering scheme. The loop yields a saturation flux density (*B_s_*) of 500 mT, a remanence (*B_r_*) of 66 mT, and a coercivity (*H_c_*) of 12 A/m. The low *H_c_* together with the narrow loop indicates good magnetic softness and a reduced field requirement for magnetisation reversal under alternating excitation. In practical power-magnetic components, a relatively high *B_s_* is beneficial because it provides higher usable flux swing before approaching saturation, which supports higher power density and improves tolerance to transient over-excitation. The modest *B_r_* is also advantageous for high-frequency operation, as it reduces residual magnetisation and helps mitigate DC-bias sensitivity and waveform distortion, thereby contributing to more stable inductance and lower hysteresis-related dissipation [33,34,35]. For completeness, the hysteresis parameters (*B_s_*, *H_c_*, and *B_r_*) of all L9 samples (E1–E9) measured using the same protocol are summarised in Table S8, allowing comparison across the processing window.

### 3.5. Sintering Mechanism

Magnetic and loss properties of MnZn ferrites are closely tied to phase evolution, densification, and microstructure development during sintering. The heating stage mainly removes volatile/organic species and advances solid-state reactions, whereas the high temperature holding stage governs densification and grain growth under controlled oxygen partial pressure. The orthogonal screening above identifies oxygen partial pressure as the dominant factor, followed by temperature and then holding time, based on a lateral comparison across the L9 set (E1–E9). However, such a main-effects analysis does not directly reveal how phase formation, densification and microstructure evolve along the sintering process under a given atmosphere-temperature schedule. Therefore, a step-sintering verification was conducted by decomposing the optimised scheme into key temperature/time nodes. This process-path analysis provides mechanistic support for the orthogonal conclusions and helps rationalise why temperature-driven phase formation and densification exert a stronger influence than holding time adjustments within the investigated range. From an implementation perspective, the step-sintering strategy is compatible with industrial programmable furnaces (batch kilns or multi-zone continuous furnaces), as it mainly requires a multi-stage temperature-time profile with controlled atmosphere setpoints. Scale-up would primarily involve ensuring temperature/atmosphere uniformity across larger thermal masses and may require modest re-tuning of ramp/soak times, which can be addressed through pilot trials and standard process monitoring.

**Figure 11:**
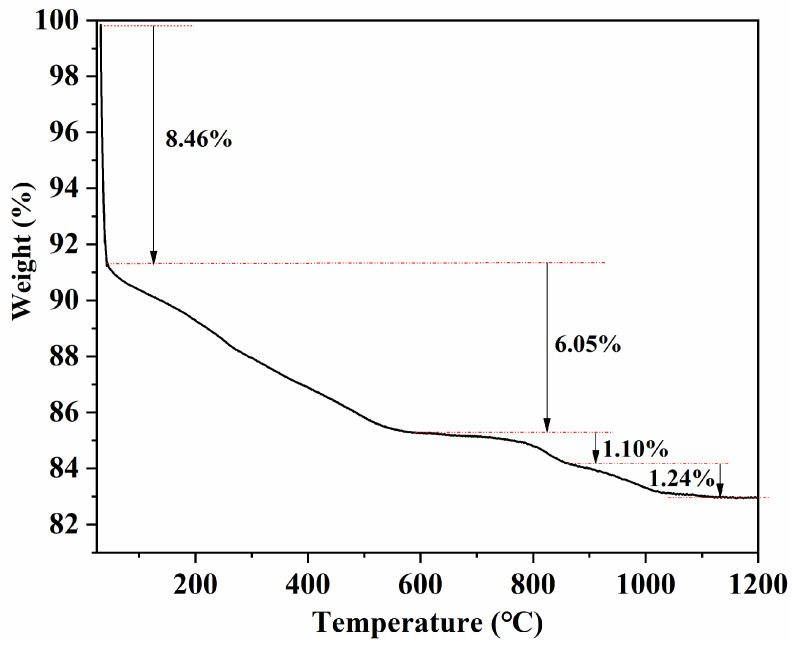
TGA curve (weight vs. temperature) of the MnZn ferrite sample measured from room temperature to 1200 °C.

To support the discussion on thermal events and possible reactions during sintering, thermogravimetric analysis was conducted from room temperature to 1200 °C (Figure 11). The sample exhibits a total mass loss of ~16.85%, which can be divided into four stages as indicated in Figure 11: an initial rapid loss of ~8.46% at low temperature, followed by a gradual loss of ~6.05% up to ~800 °C, and two minor mass-loss steps of ~1.10% and ~1.24% at higher temperatures. The low-to-intermediate temperature mass loss is mainly associated with the removal/decomposition of volatile components (e.g., adsorbed species and organic residues introduced during granulation/pressing). The minor losses at elevated temperatures may be related to further thermally activated processes, such as oxygen non-stoichiometry adjustment and/or slight volatilisation. Notably, the mass becomes nearly stable above ~1100 °C, suggesting that the major mass-change events are largely completed before entering the final high-temperature sintering window. Although the TGA measurement was limited to 1200 °C and does not fully cover the highest sintering temperature used in this work, the near-constant mass at the upper end of the curve indicates that additional mass change above 1200 °C is expected to be limited.

**Figure 12:**
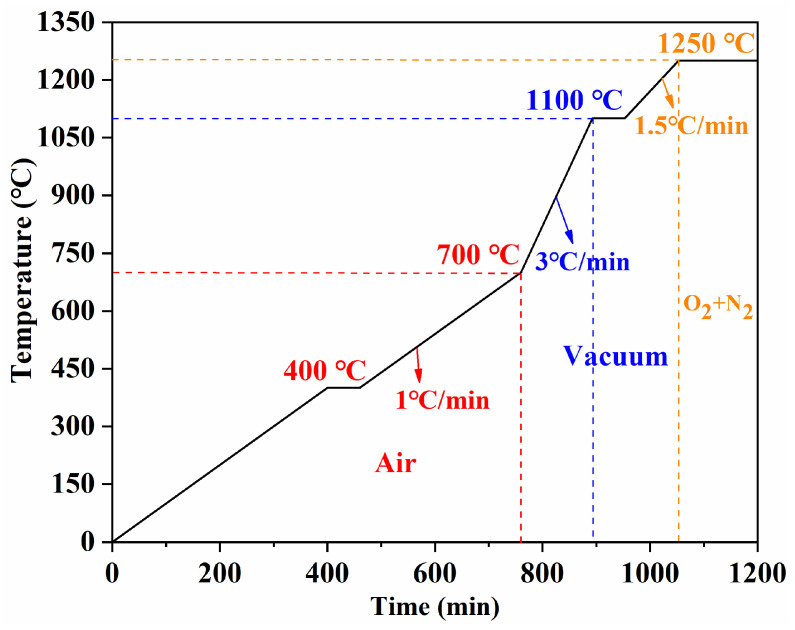
Stepwise sintering schedule based on the optimised sintering scheme.

A stepwise sintering experiment was conducted to investigate the microstructural evolution and physicochemical changes throughout the entire sintering process. Figure 12 shows the optimised sintering scheme used as the reference condition. Green toroidal compacts were heated to 400 °C, 700 °C, 900 °C, 1000 °C, 1100 °C, and 1250 °C as key sampling temperatures, and additional specimens were held at 1250 °C for different times (1 h and 3 h). The corresponding samples were denoted as S-400, S-700, S-900, S-1000, S-1100, S-1250, S-1250-1, and S-1250-3, respectively.

Taking S-400 as an example, the toroidal samples were heated in air at a rate of 1 °C/min to 400 °C. Upon reaching the set temperature, the run was terminated without an isothermal hold, and the samples were allowed to cool naturally to approximately 200 °C and removed from the furnace. This procedure was repeated for each designated sintering stage. The variations in mass loss, shrinkage, density, and initial permeability at each stage were systematically measured and are summarised in Table 4.

**Figure 13:**
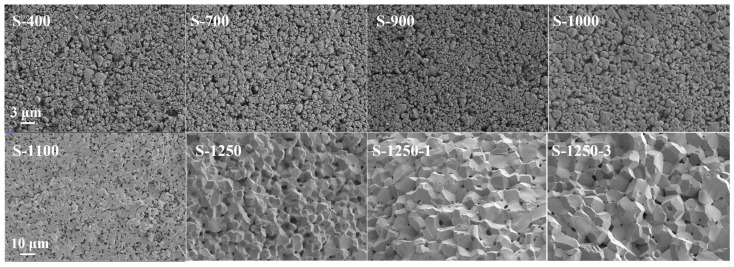
SEM micrographs of MnZn ferrite samples collected at different temperature stages during stepwise sintering.

The physical changes during sintering generally involve neck formation and densification, followed by grain growth [36]. The evolution of particle contacts, sintering necks, pores, and the formation of spinel ferrite grains in MnZn ferrite samples at different sintering temperatures can be clearly observed in the SEM pictures shown in Figure 13.

Sintering shrinkage (linear shrinkage). The bulk density and porosity of MnZn ferrites gradually improved with increasing linear shrinkage and mass loss. In the early stage of sintering, as shown in Figure 13 (S-400), the solid particles were only in initial contact, and the pores were widely dispersed. As indicated in Table 4, physically adsorbed/bound water and organic additives were removed when the temperature increased to about 700 °C, leading to reduced porosity, an increased linear shrinkage rate, and a slight rise in bulk density from 3.370 g/cm^3^ to 3.442 g/cm^3^.

In the middle stage of sintering, with further temperature increase, the interfaces between particles gradually merged, and the pores progressively closed, as observed in Figure 13 (S-900, S-1000, and S-1100). The porosity decreased rapidly, while the linear shrinkage rate significantly increased to about 8% (relative to the green compact).

At the late stage of sintering, continued heating caused closed pores to contract further, and the bulk density increased substantially, reaching about 4.8 g/cm^3^, indicating an advanced densification state. Overall, the total linear shrinkage of the ferrites exceeded 12%, as listed in Table 4.

Polycrystalline grain growth. Before sintering, the solid particles in the powders were bonded by binder/adhesive additives to maintain a certain shape. As shown in Figure 13 (S-400, S-700), during the early stage of sintering, some particles began to form fine crystalline domains, and the interparticle spacing gradually narrowed due to the volatilisation of organic residues and partial solid-phase reactions.

With increasing temperature, these microcrystals progressively merged to form sintering necks, as illustrated in Figure 13 (S-1100), which is indicative of enhanced mass transport across particle contacts and grain-boundary diffusion. In the later stage of sintering, as the temperature further increased, the neck regions shrank significantly, while the grain size of the spinel crystals increased steadily, and the grain boundaries became more distinct. When the temperature reached approximately 1250 °C, the neck features became much less discernible, and the microstructure appeared uniform and complete, as observed in Figure 13 (S-1250).

During the holding stage, the residual pores further diminished, and the grains continued to grow, as shown in Figure 13 (S-1250-1 and S-1250-3). Notably, throughout the entire sintering process, the magnetic properties of the samples gradually emerged, as evidenced by the measured permeability, which can be attributed to the progressive formation of a continuous ferrite spinel network and the accompanying grain growth that reduces domain-wall pinning.

**Figure 14:**
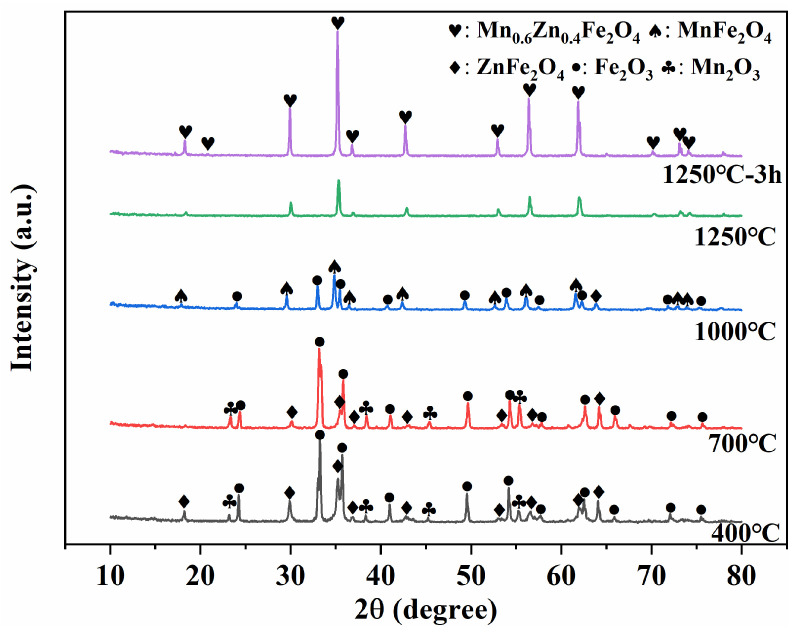
XRD patterns of samples collected at different temperature stages during the stepwise sintering experiment.

The phase composition of the samples obtained from the step-sintering experiment was identified by XRD, and the results are presented in Figure 14. At the initial stage, Fe_2_O_3_, Mn_2_O_3_, and ZnFe_2_O_4_ phases were detected, which were nearly identical to those of the raw materials shown in Figure 1b. Within the detection limit of XRD, no additional crystalline phases were observed at this stage, indicating that the material remained dominated by the pre-reacted/starting oxide components. This phase constitution is consistent with the SEM observation (Figure 13), where particles are mainly in initial contact with limited neck development.

When the temperature was raised to 700 °C, the same phases were identified; however, the peak intensity of Fe_2_O_3_ exhibited a slight decrease compared with that at 400 °C. This can be attributed to enhanced ion diffusion at elevated temperatures, which increased the number of ions migrating between components and facilitated the participation of Fe_2_O_3_ in phase transformation. Previous studies have reported that zinc ferrites (ZnFe_2_O_4_) are typically formed at around 700 °C via Reaction (2) [37]. The presence of ZnFe_2_O_4_ already at early stages in this work is attributed to the pre-firing step used in the commercial powder processing, which can alleviate the abnormal expansion associated with in situ ZnFe_2_O_4_ formation and promote subsequent ferrite phase development:

ZnO+Fe_2_O_3_ → ZnFe_2_O_4_. (2)

With the continuous increase in temperature, the solid-phase reactions proceeded through ion diffusion. Grain growth was promoted, and lattice defects were progressively eliminated, giving rise to a normal spinel structure. At ~1000 °C (S-1000), Mn-Fe spinel-related reflections became evident, indicating the formation of MnFe_2_O_4_-type ferrite according to Reaction (3), although the presence of residual Fe_2_O_3_ suggested that the reaction was incomplete owing to insufficient temperature:

4MnO + 4Fe_2_O_3_ → 4MnFe_2_O_4_. (3)

When the samples were sintered at 1250 °C, XRD analysis showed that the dominant phase evolved into Mn_1-x_Zn_x_Fe_2_O_4_ spinel, consistent with the formation of a Mn-Zn ferrite solid solution through interdiffusion and spinel incorporation of MnFe_2_O_4_ and ZnFe_2_O_4_, as described by Reaction (4) [38,39]. With extended sintering time, the diffraction peak intensities became sharper and more intense, indicating improved crystallinity, increased grain size, and reduced porosity and defects. These findings were consistent with the microstructural features observed in the SEM images (Figure 13), where densification and grain growth become more pronounced at the high-temperature and holding stages.

(1−x) MnFe_2_O_4_ + xZnFe_2_O_4_ → Mn_1−x_Zn_x_Fe_2_O_4_ (4)

**Figure 15:**
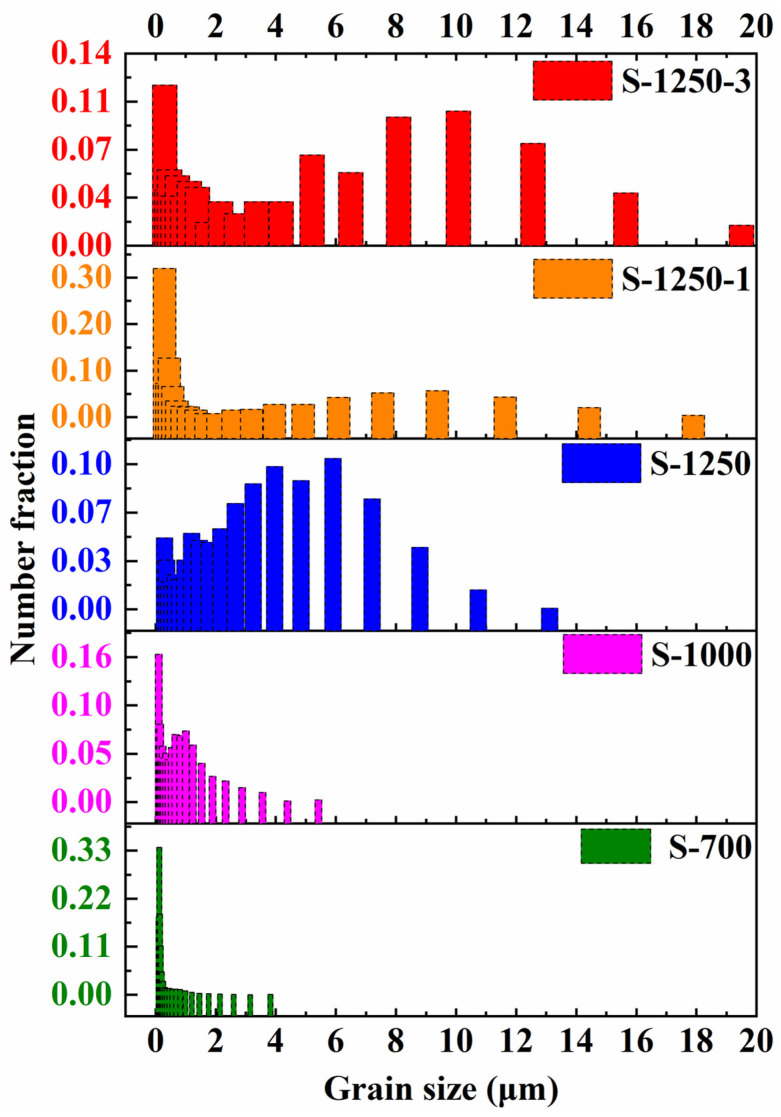
Grain size distribution (area fraction) determined by EBSD for samples collected at different step-sintering stages.

The EBSD analysis provides quantitative information on grain size and distribution, grain boundary characteristics, crystallographic orientations (texture), and phase identification [40,41,42]. It should be stated that no EBSD results were obtained for the sample sintered at 400 °C, as the specimen was not sufficiently dense and tended to crumble during mechanical polishing, which is a prerequisite for EBSD characterisation. Consistent with the SEM observations (Figure 15), a remarkable difference in grain size and distribution was observed with increasing sintering temperature. For the sample sintered at 700 °C, the grain size was mainly concentrated below 2 µm. At 1000 °C, most grains remained under 2 µm; however, the fraction of extremely fine grains decreased, with more grains distributed over a broader size range. At 1250 °C, the average grain size increased markedly, with most grains falling within the range of 2–8 µm. During the subsequent holding stage, continuous grain growth occurred, and the disparity between small and large grains became more pronounced. With an extended holding time of 3 h, grains ranging from 5 to 13 µm accounted for a larger proportion as compared with those in the S-1250-1 sample, indicating further coarsening and microstructural homogenisation as sintering proceeds.

**Figure 16:**
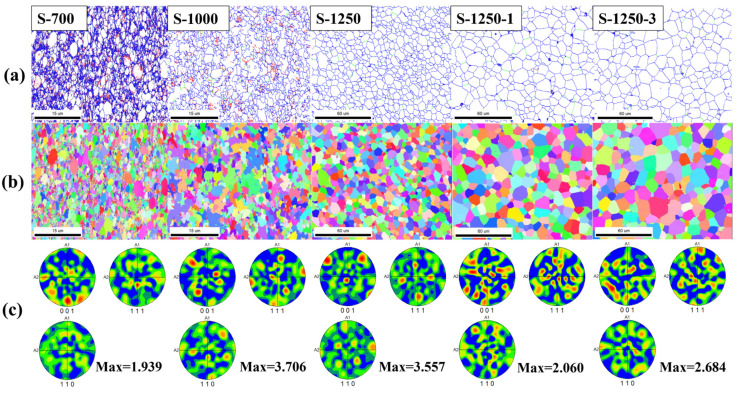
EBSD results of MnZn ferrite samples collected at different stages of the step-sintering experiment: (**a**) grain boundary map, (**b**) inverse pole figure (IPF) orientation maps, and (**c**) pole figures (PFs).

Figure 16 illustrates the microstructural evolution of samples subjected to the step-sintering experiment from a complementary perspective. Between 700 °C and 1250 °C, the process involved particle contact, merging, and the subsequent formation of new phases and grains. The microstructure evolved from a disordered state at 700 °C (S-700) to a more uniform configuration at 1250 °C (S-1250), with well-defined grain boundaries and improved grain morphology. This evolution is consistent with curvature-driven grain-boundary migration, where the reduction of total grain-boundary area/curvature provides the driving force for grain growth, leading to an overall decrease in grain-boundary energy. Furthermore, extended holding time promoted the exclusion of pores, as evidenced by the comparison between S-1250-1 and S-1250-3.

The inverse pole figures (Figure 16b) reveal the grain orientation distribution at different sintering stages. The colour follows the standard IPF colour key, where red and orange grains correspond to the (001) orientation, blue to (111), and green to (101). The orientation in S-700 appeared more complex than in S-1250-3, which is consistent with the higher microstructural heterogeneity at the early stage, compared with the predominantly single-spinel ferrite microstructure in S-1250-3. Additionally, the pole figures (Figure 16c) show no obvious symmetry, and the maximum intensity values were below four, suggesting weak texture and predominantly random grain orientations, typical of polycrystalline ferrites [43].

**Figure 17:**
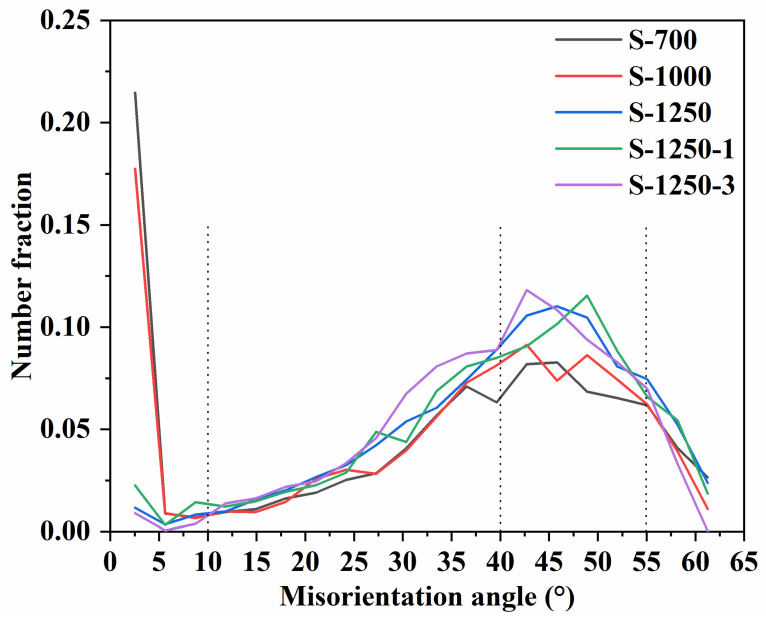
Misorientation-angle distributions of MnZn ferrite samples collected at different step-sintering stages (S-700, S-1000, S-1250, S-1250-1, S-1250-3).

Figure 17 presents the misorientation angle distribution of samples sintered at different temperatures (S-700, S-1000, S-1250, S-1250-1, and S-1250-3). The samples at the intermediate stages (S-700 and S-1000) exhibited a high proportion of low-angle grain boundaries, with a pronounced peak below 10°, accompanied by several high-angle grain boundaries with peaks in the range of 40–55°. The predominance of low-angle grain boundaries at S-700/S-1000 is more reasonably associated with a sub-grain/dislocation-rich microstructure, rather than being directly assigned to a specific residual phase. Such low-angle boundaries are commonly related to dislocation arrays and therefore reflect higher stored strain energy. With increasing temperature, recovery and grain-boundary migration can reduce dislocation density and internal stress, thereby lowering the total interfacial energy and promoting microstructural stabilisation. Conversely, the existence of high-angle grain boundaries indicates the development of more mature grain boundaries as the ferrite microstructure progressively forms and coarsens, which agrees with the phase evolution identified by XRD in Figure 14 and the microstructural observations in Figure 16 (especially the IPF maps in Figure 16b, where S-700 and S-1000 are finer and more fragmented, while S-1250-3 exhibits markedly coarser grains) [44].

At higher temperatures, ferrite grains gradually formed, and similar misorientation angle profiles were observed for S-1250, S-1250-1, and S-1250-3, all showing strong peaks in the 40–55° range. This similarity implies that the phase compositions of these samples were essentially the same. More specifically, the consistent dominance of the 40–55° HAGB peak suggests that the grain-boundary character distribution becomes relatively stable once the predominant ferrite spinel microstructure is established. However, during the holding stage, grain growth, pore elimination and microstructural coarsening occurred to further stabilise the system. As a result, variations in grain size, grain boundary characteristics, and morphology were evident among S-1250, S-1250-1, and S-1250-3, which explains why the curves were not completely identical. To complement the above microstructural evidence, grain-growth kinetics were further evaluated using the TPRE model [45,46]. The fit yields n ≈ 1.81 and an apparent Q ≈ 200 kJ/mol [47,48], which are reported in the Supplementary Information as preliminary estimates due to the limited data points.

## 4. Conclusions

A practical sintering-property map for MnZn ferrites under the targeted operating conditions was established via an L9 orthogonal design with verification characterisation. Within the investigated parameter window, oxygen partial pressure is the dominant factor for both initial permeability and power loss, followed by sintering temperature, whereas holding time shows the weakest effect. Two sintering windows were identified: the *µ_i_*-maximising condition (1250 °C/3.5% $P_{O_{2}}$/4 h) yields *µ_i_* of 3453 and *P_cv_* of 466 mW/cm^3^ at 100 kHz/200 mT, while the *P_cv_*-minimising condition (1250 °C/5% $P_{O_{2}}$/3.5 h) produces *µ_i_* of 2678 with *P_cv_* of 400 mW/cm^3^ at 100 kHz/200 mT and 182 mW/cm^3^ at 500 kHz/50 mT. The process-structure-property links were strengthened by complementary, quantitative microstructure and magnetic loss evidence, and step-sintering experiments provide mechanistic support by clarifying phase and microstructure evolution along the process path. Overall, this work demonstrates an efficient route for sintering-parameter optimisation and offers a transferable workflow for balancing *µ_i_* and *P_cv_* via window-based process selection. The quantitative optimum levels reported here are specific to the investigated MnZn ferrite composition and processing window. For other formulations, the same orthogonal-screening workflow can be applied to re-identify the optimal sintering window.

## Figures and Tables

**Table 1:** Sintering process orthogonal experiment. Each condition was prepared twice. Measurements were repeated three times on the same specimen, and the reported *µ_i_* and *P_cv_* values are averages of repeated measurements.

No.	A: Temperature (°C)	B: Oxygen Partial Pressure (%)	C: Time (h)
E1	1 (1250)	1 (5)	1 (3)
E2	1 (1250)	2 (2)	2 (3.5)
E3	1 (1250)	3 (3.5)	3 (4)
E4	2 (1280)	1 (5)	2 (3.5)
E5	2 (1280)	2 (2)	3 (4)
E6	2 (1280)	3 (3.5)	1 (3)
E7	3 (1310)	1 (5)	3 (4)
E8	3 (1310)	2 (2)	1 (3)
E9	3 (1310)	3 (3.5)	2 (3.5)

**Table 2:** Orthogonal experiment results and analysis for initial permeability.

No.	A: Temperature (°C)	B: Oxygen Partial Pressure (%)	C: Time (h)	*µ_i_*
E1	1 (1250)	1 (5)	1 (3)	2697
E2	1 (1250)	2 (2)	2 (3.5)	1750
E3	1 (1250)	3 (3.5)	3 (4)	3453
E4	2 (1280)	1 (5)	2 (3.5)	2602
E5	2 (1280)	2 (2)	3 (4)	1643
E6	2 (1280)	3 (3.5)	1 (3)	3065
E7	3 (1310)	1 (5)	3 (4)	2263
E8	3 (1310)	2 (2)	1 (3)	1269
E9	3 (1310)	3 (3.5)	2 (3.5)	2327
*K*_1_	2633	2520	2343	
*K*_2_	2436	1554	2226	
*K*_3_	1953	2948	2453	
*R*	680	1394	226	

**Table 3:** Orthogonal experiments results and analysis for *P_cv_* at 100 kHz/200 mT.

No.	A: Temperature (°C)	B: Oxygen Partial Pressure (%)	C: Time (h)	*P_cv_* (mW/cm^3^)
E1	1(1250)	1(5)	1(3)	438
E2	1(1250)	2(2)	2(3.5)	510
E3	1(1250)	3(3.5)	3(4)	466
E4	2(1280)	1(5)	2(3.5)	436
E5	2(1280)	2(2)	3(4)	877
E6	2(1280)	3(3.5)	1(3)	670
E7	3(1310)	1(5)	3(4)	568
E8	3(1310)	2(2)	1(3)	1269
E9	3(1310)	3(3.5)	2(3.5)	653
*K*_1_	471	480	792	
*K*_2_	661	885	533	
*K*_3_	830	596	637	
*R*	358	404	259	

**Table 4:** Evolution of mass loss, linear shrinkage, bulk density, and initial permeability of MnZn ferrites during stepwise sintering.

Performance	Green Body	S-400	S-700	S-900	S-1000	S-1100	S-1250	S-1250-1	S-1250-3
Mass loss rate (%)	0	−0.03	−0.32	−0.77	−0.98	−1.32	−3.08	−3.35	−3.80
Shrinkage (%)	0	0	−0.54	−1.49	−3.03	−8.28	−10.07	−12.11	−12.19
Density (g/cm^3^)	3.370	3.390	3.442	3.525	4.025	4.360	4.521	4.842	4.859
Permeability	-	-	-	-	-	541	616	1846	2678

## Data Availability

The original contributions presented in this study are included in the article/supplementary material. Further inquiries can be directed to the corresponding authors.

## Supplementary Materials

The following supporting information can be downloaded at: https://www.mdpi.com/article/10.3390/ma19040779/s1, Figure S1: XRD patterns of MnZn ferrites (E1–E9) prepared under the L9 orthogonal sintering design, Figure S2: Linearised fittings for the preliminary TPRE kinetics analysis: (a) *ln D* versus *1/T* at fixed holding time (*t* = 3 h, Table S9); (b) *ln D* versus *ln t* at fixed temperature (1250 °C, Table S10). The red lines represent linear regressions; fitting coefficients and *R*^2^ values are summarised in Table S11, Table S1: Chemical composition and key supplier specifications of the starting commercial powders (GP95), Table S2: Preliminary experiments on sintering temperature, Table S3: ANOVA (main-effects) for *µ_i_* (L9 orthogonal design), Table S4: Orthogonal experiments results and analysis for *P_cv_* at 500 kHz/50 mT, Table S5: ANOVA (main-effects) for *P_cv_* at 100 kHz/200 mT (L9 orthogonal design), Table S6: Sintering parameters and magnetic performance of MnZn ferrites, Table S7: Reproducibility of the optimised low-loss sintering condition (1250 °C, 5% $P_{O_{2}}$, and 3.5 h, duplicate preparation, test condition: 100 kHz/200 mT), Table S8: *B_s_*, *H_c_*, *B_r_* of MnZn ferrites sintered in orthogonal experiment, Table S9: Average grain size of samples sintered at different temperatures with the same holding time (*t* = 3 h), Table S10: Average grain size of samples sintered at 1250 °C with different holding time, Table S11: Linear fitting results for the preliminary TPRE kinetics analysis.
