# Supplementary material for: Orthogonal Design Optimisation of the Sintering Process for MnZn Ferrites with Step-Sintering Verification
Source: Materials (Basel). 2026 Feb 16;19(4):779. doi: 10.3390/ma19040779 (PMC12941652; doi:10.3390/ma19040779)
Supplement: Supplementary file 1 [file materials-19-00779-s001.zip › materials-4140910-supplementary.pdf]

materials

MDPI

Supplementary Materials

Orthogonal Design Optimisation of the Sintering Process for MnZn Ferrites with Step-Sintering Verification

Mengrui Li [1,2,†], Shuyu Sun [1,†], Boon Xian Chai [2], Yuqi Wang [2], M. Akbar Rhamdhani [2], Shanqing Xu [2,*] and Li Wang [1,3,*]

[1] School of Mechanical, Electrical and Information Engineering, Shandong University, Weihai 264209, China; maureenli@swin.edu.au (M.L.); 202337667@mail.sdu.edu.cn (S.S.)
[2] School of Engineering, Swinburne University of Technology, Hawthorn, VIC 3122, Australia
[3] School of Electromechanical and Automotive Engineering, Yantai University, Yantai 264005, China
* Correspondence: sxu@swin.edu.au (S.X.); wanglihxf@sdu.edu.cn (L.W.)
† These authors contributed equally to this work.

Academic Editor: Mattia Biesuz

Received: 23 January 2026
Revised: 10 February 2026
Accepted: 14 February 2026
Published: 16 February 2026

Copyright: © 2026 by the authors. Licensee MDPI, Basel, Switzerland. This article is an open access article distributed under the terms and conditions of the Creative Commons Attribution (CC BY) license.

Table S1. Chemical composition and key supplier specifications of the starting commercial powders (GP95).

Item	Value
Supplier	Nantong Guanyouda Magnet Co., Ltd. (GYD), Jiangsu, China
Product model	GP95
Supplier-reported pre-firing	800–1050 °C
Powder form/morphology	Spray-granulated near-spherical granules (Figure 2)
Particle size (approx.)	38–75 µm (Figure 2)
Fe (wt%, ICP)	40.16
Mn (wt%, ICP)	17.99
Zn (wt%, ICP)	4.62
Co (wt%, ICP)	0.22
Ta (wt%, ICP)	0.07

Table S2. Preliminary experiments on sintering temperature.

Sintering temperature (°C)	μ_i	P_{cv} (mW/cm^3) at 100 kHz/200 mT
1200	1443	1900
1350	2670	958

Figure S1 shows the XRD patterns of MnZn ferrite samples E1–E9 obtained from the L9 orthogonal design. All samples exhibit diffraction peaks characteristic of a cubic spinel

structure, indexed reflections such as (111), (220), (311), (400), (422), (511), and (440), with no obvious secondary phases detected within the XRD detection limit. These patterns were used for peak-position-based estimation of the lattice parameter *a* in the Section 3.1.

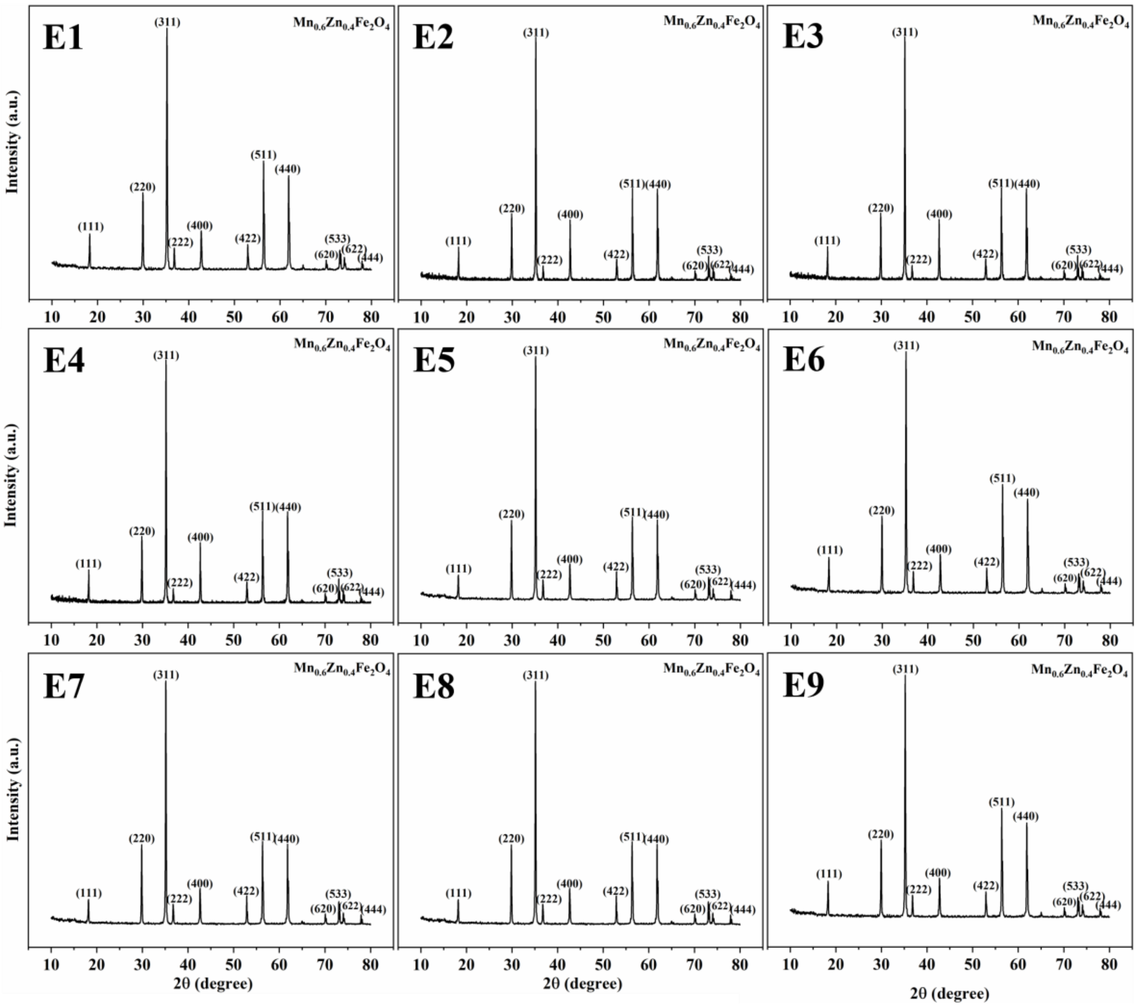

Figure S1. XRD patterns of MnZn ferrites (E1–E9) prepared under the L9 orthogonal sintering design.

Table S3. ANOVA (main-effects) for μ_i (L9 orthogonal design).

Source	DOF	SS	MS	F	Contribution (%)
A: Temperature	2	735,465	367,732	10.01	18.63
B: Oxygen partial pressure	2	3,061,509	1,530,754	41.68	77.56
C: Holding time	2	77,099	38,549	1.05	1.95
Error	2	73,454	36,727	—	1.86
Total	8	3,947,526	—	—	100.00

https://doi.org/10.3390/ma19040779

Table S4. Orthogonal experiments results and analysis for P_{cv} at 500 kHz/50 mT.

No.	A: temperature (°C)	B: oxygen partial pressure (%)	C: time (h)	P_{cv} (mW/cm³)
E1	1(1250)	1(5)	1(3)	196
E2	1(1250)	2(2)	2(3.5)	247
E3	1(1250)	3(3.5)	3(4)	215
E4	2(1280)	1(5)	2(3.5)	217
E5	2(1280)	2(2)	3(4)	319
E6	2(1280)	3(3.5)	1(3)	274
E7	3(1310)	1(5)	3(4)	287
E8	3(1310)	2(2)	1(3)	631
E9	3(1310)	3(3.5)	2(3.5)	328
K_1	219	233	367	
K_2	270	399	264	
K_3	415	272	274	
R	196	166	103	

Table S5. ANOVA (main-effects) for P_{cv} at 100 kHz/200 mT (L9 orthogonal design).

Source	DOF	SS	MS	F	Contribution (%)
A: Temperature	2	193,176.2	96,588.1	6.47	32.97
B: Oxygen partial pressure	2	260,654.9	130,327.4	8.74	44.49
C: Holding time	2	102,198.2	51,099.1	3.43	17.44
Error	2	29,837.6	14,918.8	—	5.09
Total	8	585,866.9	—	—	100.00

Table S6. Sintering parameters and magnetic performance of MnZn ferrites.

Sintering parameters		Magnetic performance	
**Temperature**	1230°C	μ_i	2547
P_{O_2}	5%	P_{cv} **(100 kHz/200 mT)**	217 mW/cm³
**Time**	3.5 h	P_{cv} **(500 kHz/50 mT)**	313 mW/cm³

Table S7. Reproducibility of the optimised low-loss sintering condition (1250 °C, 5% P_{O_2}, and 3.5 h, duplicate preparation, test condition: 100kHz/200mT).

Optimised condition	μ_i (Run 1)	μ_i (Run 2)	μ_i (Mean±SD)	P_{cv} (Run 1)	P_{cv} (Run 2)	P_{cv} (Mean±SD)
Low-loss optimum	2678	2594	2636 ± 59	400	386	393 ± 10

*The optimised condition was prepared twice (Run 1 and Run 2). For each specimen, magnetic measurements were repeated three times and the reported values are averages. P_{cv} is reported in mW/cm³). Duplicate preparation show ~3.2% variation in μ_i and ~3.6% variation in P_{cv} (100 kH/200 mT), indicating good reproducibility.

Table S8. B_s, H_c, B_r of MnZn ferrites sintered in orthogonal experiment.

No.	B_s (mT)	H_c (A/m)	B_r (mT)
E1	463	13	71
E2	495	19	80
E3	465	12	99
E4	485	13	60
E5	436	17	59
E6	501	14	83
E7	459	15	78
E8	474	13	151

https://doi.org/10.3390/ma19040779

E9	465	19	79

To illustrate the grain-growth behaviour of MnZn ferrites, representative samples from the orthogonal design were selected: E1, E2, E3 (varying sintering temperature at a fixed holding time) and E1, E5, E7 (varying holding time at a fixed sintering temperature). The corresponding average grain sizes are listed in the Table S9 and Table S10. The grain growth kinetics was analysed using the Traditional Phenomenological Rate Equation (TPRE):

$$D^n - D_0^n = K_0 \cdot t \cdot e^{-Q/RT} \quad \text{(S1)}$$

where D is the average grain size at holding time t, D_0 is the initial average grain size, n is the grain-growth exponent, Q is the apparent activation energy, T is the absolute temperature (K), K_0 is a constant, R is the gas constant. In the present sintering regime, $D_0 \ll D$ is assumed, thus, the Equation (S1) can be approximated as:

$$D^n = K_0 t e^{-Q/RT} \quad \text{(S2)}$$

Taking the natural logarithm yields:

$$lnD = lnt/n - Q/nRT + lnK_0/n \quad \text{(S3)}$$

At fixed T (1250 °C), n was preliminarily estimated from the slope of the *ln D*-*ln t* linear fit (Table S10). At fixed t (3 h), the apparent Q was obtained from the slope of the *ln D*-*1/T* linear fit (Table S9) combined with the estimated n. The fitting equations, slope/intercept values, and goodness-of-fit metrics (R^2) are summarised in Table S11, and the corresponding linearised plots are shown in Figure S2.

Based on the present datasheets, the exponent is preliminarily estimated as $n \approx 1.81$ and the apparent activation energy as $Q \approx 200$ kJ/mol. Within the investigated range, the estimated exponent is close to the classical parabolic growth behaviour ($n \approx 2$), which is consistent with grain growth dominated by grain-boundary migration [47, 48]. It should be noted that this analysis is based on only three temperature points and three time points, therefore, the obtained n and Q values should be interpreted as preliminary/apparent estimates. A more reliable determination requires additional temperature/time points and repeated grain-size statistics with error bars.

Table S9. Average grain size of samples sintered at different temperatures with the same holding time (t = 3 h).

***T* (K)**	**1523.15**	**1553.15**	**1583.15**
***D* (μm)**	10.35	12.26	14.4

Table S10. Average grain size of samples sintered at 1250 °C with different holding time.

***t* (h)**	**3**	**3.5**	**4**
***D* (μm)**	10.35	10.94	12.15

Table S11. Linear fitting results for the preliminary TPRE kinetics analysis.

**Fitting (x, y)**	**Linear equation**	**Slope**	**Intercept**	R^2
x = *ln t*, y = *ln D* (fixed T = 1250 °C)	*ln D* = *m ln t* + *b*	*m* = 0.5523 ± 0.1221	*b* = 1.7208 ± 0.1528	0.9534
x = *1/T*, y = *ln D* (fixed t = 3 h)	*ln D* = *m* (*1/T*) + *b*	m = -(1.3273 × 10^4) ± 48.50	*b* = 11.0514 ± 0.03124	0.9999866

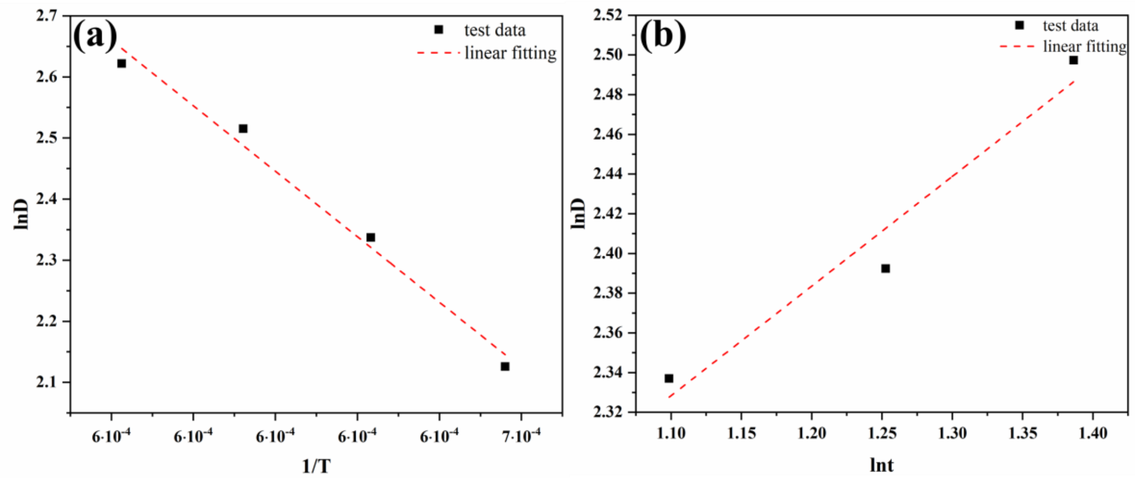

Figure S2. Linearised fittings for the preliminary TPRE kinetics analysis: (a) *ln D* versus *1/T* at fixed holding time (t = 3 h, Error! Reference source not found.); (b) *ln D* versus *ln t* at fixed temperature (1250 °C, Error! Reference source not found.). The red lines represent linear regressions; fitting coefficients and R^2 values are summarised in Error! Reference source not found..

Disclaimer/Publisher's Note: The statements, opinions and data contained in all publications are solely those of the individual author(s) and contributor(s) and not of MDPI and/or the editor(s). MDPI and/or the editor(s) disclaim responsibility for any injury to people or property resulting from any ideas, methods, instructions or products referred to in the content.

https://doi.org/10.3390/ma19040779
